# Effect of an Aquatic Balance-Training Program in Patients with Chronic Stroke: A Single-Group Experimental Pilot Study

**DOI:** 10.3390/medicina56120656

**Published:** 2020-11-28

**Authors:** Sagrario Pérez-de la Cruz

**Affiliations:** Department of Nursing, Physiotherapy and Medicine, University of Almería, 04120 Almería, Spain; spd205@ual.es; Tel.: +34-950-214-574

**Keywords:** aquatic therapy, balance, stability, stroke, Halliwick

## Abstract

*Background and Objectives*: Stroke survivors face severe problems affecting their mobility, such as balance impairments and an increased risk of falls. The principal aim of this study was to quantify the effects of 12 sessions of Halliwick aquatic therapy for the treatment of balance in patients with chronic stroke. *Materials and Methods*: Twenty-nine people with stroke participated in this single-group experimental pilot study. Sessions were performed three times a week for a total of 12 sessions. A stabilometric assessment was conducted using a computerized platform. The evaluations were performed at baseline, at four weeks, and one month after completing the aquatic program. *Results*: The results obtained revealed significant differences for postural stability values (*p* < 0.001) and single-leg stabilometry. However, in the case of values within the limits of stability, such as forward (F = 0.339, *p* = 0.676), backward (F = 0.449, *p* = 0.644), forward right oscillations (F = 1.637, *p* = 0.21), and the anterior/posterior instability index (F = 0.614, *p* = 0.55), no significant differences were found. *Conclusions*: These findings suggest that Halliwick therapy may potentially improve stroke balance impairments. The main limitations of the study were the sample size, the lack of a control group, and the study not being blind.

## 1. Introduction

Stroke is one of the main causes of adult morbidity and mortality in developed countries, particularly among women. In Spain, stroke represents the second cause of specific mortality with an estimated prevalence of 120–350 cases for every 100,000 habitants/year, multiplied by 10 in populations over the age of 75 [1]. Furthermore, between 5% and 11% of the population over the age of 65 has a clinical background of stroke, highlighting its high prevalence as a cause of death, disability, and dependency, considering that 40% of survivors report some degree of functional decline [1,2].

Physical therapy and rehabilitation programs are effective for the recovery of function and mobility after a stroke [3,4]. Furthermore, research to date supports the effectiveness of treatment in water for the recovery of function [1,2,3]. Aquatic therapy, according to Alonso [5], is a therapeutic procedure based on the mechanical properties of water and is combined with specific therapeutic techniques in order to facilitate function and the attainment of therapeutic objectives. In particular, adults with neurological disorders may benefit from these techniques [6,7,8,9,10,11,12,13,14]. Several treatment approaches exist, such as Halliwick, Ai Chi, Bad Ragaz, Watsu, and hydrokinesitherapy, among others [7,8,9,10]. The Halliwick concept is a structured learning process featuring specific exercises aimed at obtaining independence in water and improving the control of movement and balance [11,12,13]. These unique characteristics may be used for the treatment of patients with stroke for whom balance deficits represent one of the greatest problems, being associated with a higher frequency of falls and disability. Ultimately, this may affect the performance of activities of daily life and restrict socialization [7,15,16,17]. Those who participate in this type of aquatic program experience first-hand the properties of exercise in this medium, such as buoyancy, water density, hydrostatic pressure, and temperature. As a result, loads are decreased and patients experience increased range of motion [15]. Furthermore, exercise performed in water temperatures above 30 °C promotes relaxation, relief from joint pain, and a decrease in muscle tone [13,16]. 

The principal aim of this study was to quantify the effects of 12 sessions of Halliwick aquatic therapy for the treatment of balance in patients with chronic stroke.

## 2. Materials and Methods 

### 2.1. Study Design and Sample

This pilot study was a single-group experimental study (PTJ-2019-0588). Twenty-nine patients with a diagnosis of non-traumatic acquired brain injury were selected based on the following inclusion criteria: (1) a medical diagnosis of stroke (certified by computerized tomography or magnetic resonance imaging); (2) >6 months post injury; (3) the ability to independently walk for a minimum of 10 m, with or without aids; and (4) preserved cognitive skills, with a score greater than or equal to 24 on the Mini-Mental State Examination [18].

The exclusion criteria were (1) presenting a disability on the modified Rankin scale [19] of four or more; (2) the presence of any associated pathology or disorder that could modify and alter the normal execution of the test, such as severe cognitive decline; and (3) any absolute contraindication for aquatic therapy, such as vestibular disorders, previous thoracic or abdominal surgery, recent myocardial infarct, decompensated cardiac insufficiency, uncontrolled hypertension, or orthostatic hypotension. 

**Figure 1:**
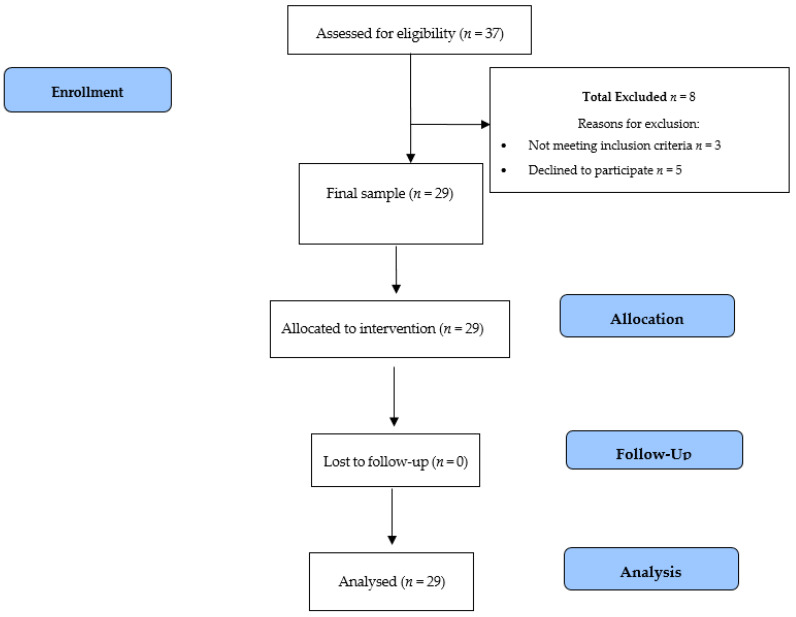
Study design flowchart.

All participants initially considered suitable for participation in this program were informed of the study and asked to provide their informed consent. This study was conducted according to the regulating standards of good clinical practice and the Helsinki Declaration (2013) and approved by the Bioethics Committee of the University of Almería (UALBIO2017/007). Figure 1 displays the selection process of the study participants.

### 2.2. Stabilometric Assessment

The stabilometric assessment was performed using a computerized platform with a 55 cm diameter (Balance System SD, Biodex Medical Systems Inc., Shirley, NY, USA) designed to test the kinesthetic ability of patients by offering 12 programs for assessing the levels of stability on the moving platform (this platform remains stationary for static measurements) [20]. For each subject, a static test was performed followed by a dynamic test. 

The limits of stability training screen is designed to challenge the user to move through a movement pattern consistent with the sway envelope. The sway envelope is the area where a person can move their center of gravity within their base of support. It is calculated from vertical as 8 degrees to one side, 8 degrees to the other (for a total of 16 degrees of oscillation in the frontal plane), and 8 degrees forward and 4 degrees back (12 degrees total in the sagittal plane). Training and testing of the limits of stability is based on challenging the patient within this sway envelope [21].

During the static test (postural stability) the patient was requested to maintain an upright vertical position on a fixed platform over a period of 20 s while the system registered the trunk oscillations of the center of mass (registered prior to the commencement of the test) in any spatial direction. 

For optimal operation, the patient must be ensured to be standing in the center of the platform. The test was repeated three times and a software program calculated the mean score, which was compared with normative data categorized by age [20,21]. The test was performed in silence to avoid distracting patients, as it has been found that cognitive influences may alter subjects’ postural control [22]. In cases where the subject wore glasses, they were used during all tests.

The next test consisted of evaluating the stability limits in dynamic conditions. The limits of stability test challenges patients to move and control their center of gravity within their base of support. During the test, the patients must move their center of mass in order to move the cursor of the central target to a blinking target and return as fast as possible and with as little deviation as possible. This process is repeated for each of the nine targets. The targets on the screen blink in a random order. Three skill levels allow the targets to be grouped further together or further apart. This test is a good indicator of dynamic control with non-normalized oscillation [20,21]. Deficient control, inconsistencies, or increased time for the performance of the same test suggest a lack of strength in the lower limbs, proprioceptive deficits, or vestibular or visual deficits.

Last, we evaluated the single-leg stabilometry. This test consists of assessing the ability of a person to stay on one leg. This test is performed on both sides for each patient [20].

### 2.3. Intervention/Aquatic Therapy

The aquatic therapy program was performed in its entirety in a pool specifically designed for the performance of aquatic therapy techniques. The sessions took place in a pool measuring 20 × 8 m, with a minimum depth of 140 cm. The water temperature was 32 °C (±2 degrees), and the room temperature was 24 °C.

During the four weeks’ duration of the study, the 29 patients received 12 aquatic therapy sessions in total (three per week), which consisted of individual interventions lasting 45 min. These were performed by a physiotherapist specialized in aquatic therapy who worked at the center where the study was performed, and who was external to the study.

The sessions were programmed with a progression in the level of difficulty. Initially, certain exercises were performed for patients to become increasingly familiarized with the water and more adapted to the environment. At end of each session, participants were taught to stretch and perform relaxation techniques in flotation [23]. 

In Halliwick, the ability to adjust to the fluid mechanics of the water environment is very important (floatability, flow conditions, waves). Breathing control is another salient aspect. The initial part of the program consisted of walking exercises in the aquatic environment as a warm-up, as well as stretching the upper and lower extremities. This was completed with trunk mobility, stabilization, and rotation exercises as the principal exercises. Participants progressed through each of the 10 steps, combined with specific basic exercises. In particular, the Halliwick concept contains complex rotation movements in which the participant is completely immersed in the water and subsequently requires significant control of the center of gravity. Subsequently, participants must train the ability to control movements around the longitudinal body axis. The rotation movements are considered especially important. Therapeutically, the most important aspect is counter rotation. Once these aspects are controlled, the therapist proposes the use of immersion with turbulence and relaxation so that the participants can continue to control the body activity in the aquatic environment. A small swimming movement using the hands is also included as a preparation for a true propulsion activity. For this purpose, it is important to have automatic trunk control. Last, a swimming propulsion movement using the arms (rowing) was proposed. Individual adaptations were allowed according to each patient’s capability [16]. Each participant only progresses if he or she has completed the original exercise independently and safely. All participants in the study progressed to the last step of Halliwick.

Table 1 below describes the intervention program used.

### 2.4. Statistical Analysis

The statistical analysis of the results obtained was performed by an expert external to the study. Three assessments were performed: pre-treatment, upon completion of the proposed program, and one month after completing the sessions. The normality of the data was confirmed using the Shapiro–Wilk test. In order to identify the effects that aquatic therapy had on the population under study, repeated ANOVA measures for related samples was performed. The statistical analysis was performed using the SPSS v. 23 statistical program. All the data fulfilled the criteria of normality; therefore, the analysis was performed via parametric statistics. A value of *p* < 0.05 was considered statistically significant (post hoc analyses were conducted and the use of Bonferroni correction adjusted the confidence level for each individual interval so that the resulting simultaneous confidence level was equal to the specified value).

## 3. Results

The sample comprised 29 patients, of whom 20 were men (68.96%) and nine were women (31.03%). Table 2 shows the socio-demographic characteristics of the study sample.

No adverse events were recorded. All patients completed the treatment, fully complying with the proposed program. No incident was registered leading to any undesired effect. Upon completion of the intervention, the results obtained indicated the existence of a series of changes in the variables under study. These data are displayed in Table 3, Table 4 and Table 5. 

The findings shown in Table 2 indicate that the results obtained in terms of stability in the different planes between the first two measurements (pre- and post-intervention), are statistically significant, whereas in relation to the third measurement (one month after the end of the therapy), the results are not statistically significant compared to those obtained upon completion of the intervention. 

The analysis of the results obtained for the principal effect (time) revealed significant differences in the progression of patients for each of the items analyzed. After four weeks of treatment, the scores for postural stability, stability limits, and single-leg stability overall significantly improved for patients in the sample (*p* < 0.01). Among the values obtained in the three measurements, it is important to point out that the results obtained in the limits of anterior and posterior stability did not display clear significant differences.

In addition, the benefits obtained in our sample were maintained over time (one month after completing the therapeutic intervention), whereas with regard to the values displayed by the limits of stability items backwards and towards the right, the changes were either not constant or the change was not maintained for the values obtained in the measurement performed upon completion of the therapy (*p* = 0.005 and *p* = 0.007, respectively).

## 4. Discussion

This study sought to quantify the effects of a program of aquatic therapy in a population of patients diagnosed with stroke. After completing the intervention, patients displayed improvements in their balance, according to the results observed in the various tests performed.

The values obtained in the assessment of the motor skills of patients with stroke are important for evaluating and predicting balance and gait skills during the motor tasks required during activities of daily life. The loss of the ability to perform motor skills can lead to a decline in autonomy and greater dependency [7]. Other studies [24,25,26,27] have reported improvements in postural control, dynamic balance, and gait speed, which were significantly greater after a program of aquatic intervention. Furthermore, a smaller number of falls was found in the treatment group compared to neurological patients receiving therapy on dry land [28,29,30].

The results of previous studies on the topic of aquatic interventions in patients with chronic stroke are similar to the current study. Noh et al. and Eyvaz et al. [28,29] both evaluated the effect of aquatic therapy in patients post stroke. The study by Noh et al. was based on a program of Ai Chi and Halliwick compared with patients who received therapy on dry land [28], whereas Eyvaz et al. [29] combined aquatic therapy and dry land therapy, which was compared with a group that did not receive aquatic therapy. In both cases, patients who performed therapy in water achieved a significant improvement in scores related to balance, whereas conventional therapy did not reveal significant benefits. The results of this study corroborate the tested hypotheses of these previous studies, in this case quantifying the changes observed on the scores of each test performed. The Halliwick method has also been examined and applied intensively to the same patient population by Morer et al. [31] in a prospective quasi-experimental study. This former study involved a specific three-week program conducted on patients with a cerebrovascular accident (CVA) and with mild to moderate disability. All the results analyzed suggest that an intensive program of aquatic therapy may also be useful during the rehabilitation of CVAs in order to obtain improvements in balance, gait, and pain. Regarding this last item, another study also examined the psychological aspect of disability in these patients, reporting an improvement in aspects such as depression and anxiety (common disorders in these patients) [32]. The intensive program carried out by this study is similar in duration to that of our patients, however, unlike these studies [31,32], we were able to quantify the values obtained with exact measurements of the stability amplitudes of each individual. This reinforces the idea of aquatic therapy as a suitable means for working with the type of patient who displays deficiencies in their static and dynamic balance. 

Another notable study was performed by Zhang et al. [33], who sought to evaluate the effects of a program of aquatic exercise designed to improve the muscle strength of the lower hemiparetic limb in patients with stroke. This study comprised 36 patients divided into two groups (a control group that received treatment on dry land, and an experimental group that received the therapy in the water. Compared with the conventional intervention, the aquatic intervention was related to significantly greater knee extension and ankle plantar flexion accompanied by significantly less knee extension in the paretic limb. The scores on the modified Ashworth scale did not reveal differences among groups. This study indicated that exercise in water improved muscle strength in the paretic lower limbs and improved muscle contraction without increasing spasticity in patients with subacute stroke. The strength work used in this program revealed that this type of therapy, with the lower limb approach, is a goal to be achieved, since the stability required for ambulation (monopodal support phase of the gait) can be achieved if the affected lower limb can maintain the load distribution necessary for correct walking and standing for a given period of time.

The results of this study concur with previous reports [15,16,17,24,30,34,35,36]. Based on these combined findings, the aquatic environment appears to be an ideal medium for the rehabilitation of balance in people who have suffered a stroke, achieving better results when compared to therapy on dry land, even in studies with different interventions or with patients at different stages of their rehabilitation. This supports the theory regarding the usefulness of aquatic therapy as being a positive environment for training balance, thanks to the stimuli produced for the patients by the water’s inherent physical properties.

The physical properties of water offer decreased body weight and increased support for weak body segments. The buoyancy of water and the metacentric effects constitute a constant challenge for balance during therapy, providing greater stimuli and eliciting balance reactions during training, which can lead to greater improvements. Therefore, it appears that aquatic therapy may achieve greater results for improvement in the static and dynamic balance of individuals, compared to therapy on dry land.

One of the limitations of this study was related to the sample. This was a non-randomized, single group trial and therefore, one cannot draw any conclusions on the effectiveness of the intervention. The intervention (Halliwick method) is difficult to standardize between participants. In future studies, it would be recommendable to increase the number of participants in order to more safely extrapolate the results obtained in this pilot study. Furthermore, this study also lacked a control group to which to compare findings and assess possible differences between treatments. It would also have been interesting to be able to re-evaluate the participants over a longer period of time after completion of therapy in order to verify the duration of the changes that occurred.

## 5. Conclusions

This pilot study found that aquatic therapy was effective for maintaining and improving balance (and, therefore, function of activities of daily life) in a sample of people with stroke, although the sample size would have to be increased and compared to a control group and the study blinded in order to confirm the results. The aquatic environment offers numerous advantages, representing a useful therapeutic environment in which to provide comprehensive rehabilitation and improvements in the function of activities of daily life.

## Figures and Tables

**Table 1:** Halliwick program.

Session Parts	Progression	Duration
Warm-Up	✓ Walking exercise ✓ Upper limb stretches ✓ Lower limb stretches	10 min
Halliwick Therapy	Transverse rotation control Sagittal rotation control Longitudinal rotation control Combined rotation control Immersion with turbulence environment Swimming propulsion movement using the arms (rowing)	25 min
Cool-Down	➢ Return to calm	10 min
Total duration of the session		45 min

**Table 2:** Demographic and clinical characteristics of the study participants.

	Participants			IQ 95%
	Male (*n* = 20)	Female (*n* = 9)	Total (*n* = 29)	
**Age (years)**	48.1 (2.86)	45.8 (2.65)	47.2 (7.07)	(42–55)
**Weight**	73.63 (9.02)	68.04 (6.89)	71.9 (14.55)	(59.44–82.76)
**Height (cm)**	177 (0.22)	172.71 (0.08)	176.01 (8.12)	(166–188)
**BMI**	23.69 (2.38)	21.54 (2.88)	22.71 (3.14)	(19.25–27.39)
**Mean time since diagnosis (years)**	4.20 (2.50)	5.56 (3.01)	4.93 (3.68)	(3.22–7.98)

Age (years), height (centimeters), weight (Kg), and body mass index (BMI; mean, standard deviation).

**Table 3:** Postural stability values.

	Measurement, *Mean (SD)*						ANOVA MR		
	Pre		Post		1 Month		*F* (2.22)	*p*-Value	eta*^2^*
**Overall Stability Index**	1.10 (0.70)	a	0.68 (0.29)	b	0.59 (0.28)	b	8.951	**0.001**	0.449
**Anterior/Posterior Instability Index**	0.88 (0.41)	a	0.52 (0.22)	b	0.43 (0.11)	b	12.269	**<0.001**	0.527
**Medial/Lateral Index**	0.65 (0.42)	a	0.38 (0.18)	b	0.41 (0.28)	b	9.599	**0.001**	0.466

Weight asymmetry (%) between the half-planes of the total group in each sling condition on static balance test (mean ± standard error); a, b: post hoc analysis. Between the two columns, different letters indicate statistically significant differences between time moments (Bonferroni correction).

**Table 4:** Limits of stability values.

	Measurement, *Mean (SD)*						ANOVA MR		
	Pre		Post		1 Month		*F* (2.22)	*p*-Value	eta*^2^*
**Time to complete trial (sec)**	79.75 (20.48)	a	54.25 (16.77)	b	512.5 (12.19)	b	24.457	**<0.001**	0.69
**Overall (mm)**	23.50 (14.79)	a	33.67 (14.90)	b	29.50 (12.18)	a	6.731	**0.005**	0.38
**Forward (mm)**	23.33 (18.57)		27.08 (16.88)		24.58 (13.94)		0.399	0.676	0.035
**Backward (mm)**	32.42 (10.60)		33.83 (15.46)		31.33 (15.41)		0.449	0.644	0.039
**Right (mm)**	35.08 (19.60)	a	51.00 (22.55)	b	45.08 (20.42)	c	6.196	**0.007**	0.36
**Left (mm)**	35.75 (20.99)	a	48.17 (18.59)	b	41.58 (14.16)	b	3.464	**0.049**	0.239
**Forward right (mm)**	33.17 (19.04)		40.25 (13.03)		40.00 (13.42)		1.675	0.21	0.132
**Forward left (mm)**	28.83 (16.53)	a	35.25 (13.87)	b	34.75 (14.98)	b	3.56	**0.046**	0.229
**Backward right (mm)**	28.25 (12.67)	a	36.25 (16.89)	b	33.33 (12.94)	b	6.171	**0.007**	0.359
**Backward left (mm)**	34.08 (11.33)	a	44.58 (19.27)	b	38.75 (14.50)	b	3.703	**0.041**	0.252

mm: millimeters; sec: seconds; a, b: post hoc analysis. Between the two columns, different letters indicate statistically significant differences between time moments (Bonferroni correction).

**Table 5:** Single leg stabilometry results.

	Measurement, *Mean (SD)*						ANOVA MR		
	Pre		Post		1 Month		*F* (2.22)	*p*-Value	eta*^2^*
**Overall Stability Index**	1.65 (0.51)	a	1.17 (0.60)	ab	1.04 (0.47)	b	5.491	0.012	0.333
**Anterior Position Index**	1.15 (0.33)		0.98 (0.45)		0.98 (0.48)		0.614	0.55	0.053
**Medial Lateral Index**	1.08 (0.28)	a	0.71 (0.41)	b	0.86 (0.41)	b	3.471	**0.049**	0.238

Weight asymmetry (%) between the half-planes of the total group in each sling condition on static balance test (mean ± standard error); a, b: post hoc analysis. Between the two columns, different letters indicate statistically significant differences between time moments (Bonferroni correction).
